# The Role of Iron‐Hyponitrite Intermediates in Biology and Insights From Synthetic Model Complexes

**DOI:** 10.1002/chem.71156

**Published:** 2026-05-29

**Authors:** Michael O. Lengel, Nicolai Lehnert

**Affiliations:** ^1^ Department of Chemistry University of Michigan Ann Arbor Michigan USA

**Keywords:** biomimetic complexes, iron complexes, nitric oxide reductases, nitric oxide, nitrogen cycle

## Abstract

Nitric oxide reductases (NORs) are a class of metalloenzymes that are known to mediate the 2 e^−^/ 2 H^+^ reduction of nitric oxide (NO) to nitrous oxide (N_2_O). There are three known types of NOR enzymes, each using a different iron‐based active site to mediate NO reduction. Despite the difference in active site architecture across NORs, NO reduction is expected to be linked by a common intermediate: hyponitrite (N_2_O_2_^2−^). However, experimentally, very little is known about the expected Fe‐hyponitrite intermediates. In this Perspective, we provide a detailed discussion of Fe‐hyponitrite chemistry. We start with an introduction into NOR metalloenzymes and provide a detailed account of the known and proposed steps of catalytic NO reduction by these enzymes with a specific focus on the proposed Fe‐hyponitrite intermediates. We then turn our focus on what is known about synthetic Fe‐hyponitrite complexes. We introduce hyponitrite as a ligand and discuss sources of preformed hyponitrite. We then introduce the three known Fe‐hyponitrite complexes and discuss how these relate to NO reduction in NORs. Finally, we summarize what is known about Fe‐hyponitrite chemistry and give an outlook on the aspects of this chemistry that are still vastly underexplored.

## Graphical abstract

Despite the difference in active site architecture, NO reduction by different NOR enzymes is expected to be linked by a common intermediate: hyponitrite (N_2_O_2_^2−^). However, experimentally, very little is known about the coordination chemistry of iron with hyponitrite and the expected Fe‐hyponitrite intermediates. In this Perspective, we provide a detailed discussion of Fe‐hyponitrite chemistry, and comparison with select Ni‐ and Cu‐hyponitrite complexes.

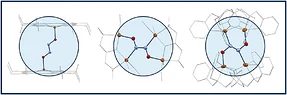

## 1 Introduction

Nitric oxide reductases (NORs) are a class of enzymes that facilitate the reduction of nitric oxide (NO) to nitrous oxide (N_2_O) according to Equation 1 [1, 2, 3]. Unlike most steps of the denitrification pathway, NO reduction is unique as it requires the formation of a new covalent bond to facilitate N_2_O formation. The proposed intermediate that is formed during this process is known as hyponitrite (N_2_O_2_^2−^). Two heme containing enzymes are known to facilitate this reactivity as part of the nitrogen cycle: Fungal NO Reductase (Cytochrome P450nor) and Bacterial NO Reductase (*c*NOR) [4, 5, 6, 7]. The reduction of NO is not exclusively part of the denitrification pathway. Some pathogens have developed enzymes that are a subclass of Flavodiiron Proteins (FDPs) called Flavodiiron Nitric Oxide Reductases (FNORs) that also reduce NO to N_2_O as NO scavengers, to circumvent the natural mammalian immune response by neutralizing NO [8, 9, 10]. Each of these Fe‐containing NORs are postulated to catalyze NO reduction differently; however, in each case, the formation of hyponitrite (N_2_O_2_^2−^) intermediate(s) is proposed upon N–N bond formation. Significant efforts have been undertaken to characterize these Fe‐hyponitrite intermediate(s) in NORs without success. Synthetic model complexes have thus far provided limited, but valuable, insights into the properties of Fe‐hyponitrite complexes and their reactivity. In this Perspective, we provide an overview of Fe‐hyponitrite chemistry in enzymes and model complexes. We first summarize proposed hyponitrite intermediate(s) involved in Cyt. P450nor, *c*NOR, and FNOR enzymes. We outline important features of these enzymes and the proposed catalytic steps of NO reduction with emphasis on the proposed hyponitrite intermediate(s). Next, we discuss the basic properties of hyponitrite salts to provide context with respect to the typical structure and behavior of hyponitrite. Finally, we summarize the current progress and provide a future outlook of Fe‐hyponitrite chemistry. We note that a thorough and comprehensive review by the Lehnert group on NOR enzymes and model complexes has been published quite recently [1]. We point the reader to this review for a more detailed description of NOR enzymes. Furthermore, Hayton and coworkers have reviewed metal‐hyponitrite coordination compounds in general [11]. In this Perspective, our efforts are focused on the formation and activation of the proposed hyponitrite species in NOR enzymes and their synthetic model complexes. We therefore focus on Fe‐hyponitrite chemistry and point the reader to the review by Hayton and coworkers for a discussion of hyponitrite complexes with other transition metals.

(1) $$2\mathrm{NO}+2e^{-}+2H^{+}\to N_{2}O+H_{2}O$$

## 2 Iron Enzymes and Hyponitrite Intermediate(s)

### 2.1 Cytochrome P450 NO Reductases (Cyt. P450nor)

**FIGURE 1:**
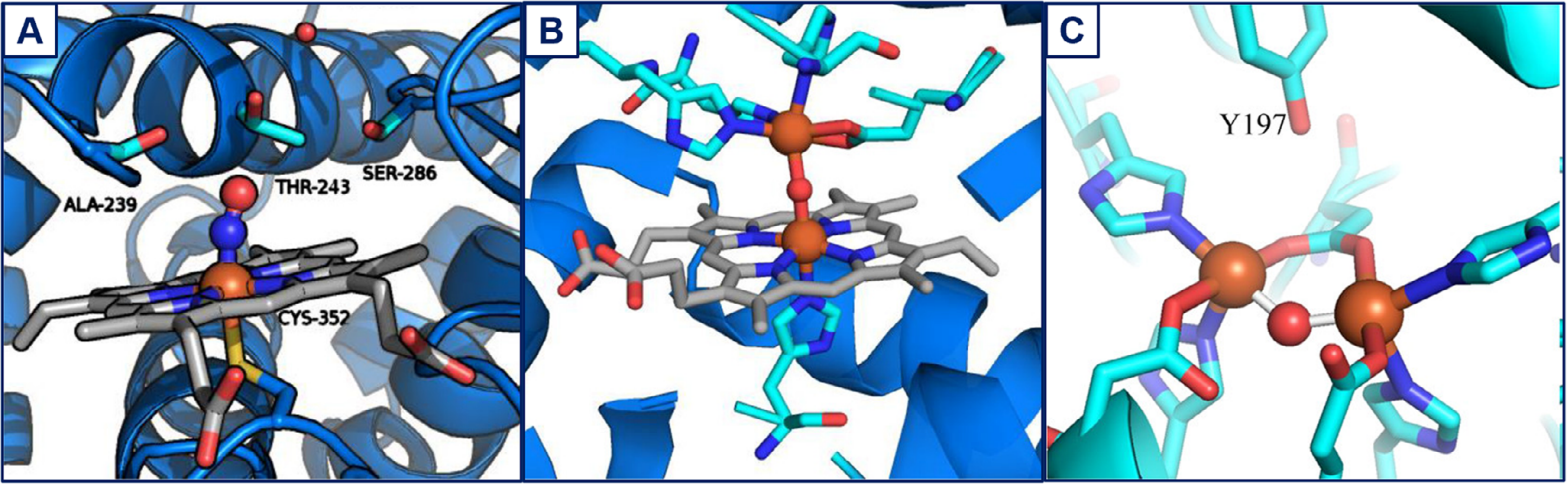
(A) PyMOL generated image of the crystal structure of the ferric active site of *Fusarium oxysporum* Cyt. P450nor with bound NO (PDB 1CL6) [18]. (B) PyMOL generated image of the crystal structure of the heme/non‐heme diiron active site of *Pseudomonas aeruginosa c*NOR in the diferric resting state (PDB 3O0R) [19]. (C) PyMOL generated image of the crystal structure of the non‐heme diiron active site of deflavinated *T. maritima* FDP, including the proposed SCS hydrogen‐bond donor Y197 (PDB: 1VME).

Cytochrome (Cyt.) P450nor is a class of fungal enzymes that belongs to the Cyt. P450 superfamily and is primarily found in yeasts and soil dwelling fungi [12, 13]. The majority of Cyt. P450 enzymes perform oxidative chemistry [14]. Therefore, it is quite surprising that Cyt. P450nor is unable to mediate typical Cyt. P450 monooxygenase chemistry and instead, catalyzes the reduction of NO to N_2_O [6, 12, 13]. Fungal denitrification ends with the formation of N_2_O (and not N_2_), suggesting that these enzymes may have a more protective function against NO toxicity [13, 15, 16]. The active site of Cyt. P450nor features a single heme *b* cofactor with a proximally bound cysteinate residue (see Figure 1A) [17, 18], which has been postulated to be a key factor in the ability of this enzyme to facilitate NO reduction [1].

**SCHEME 1:**
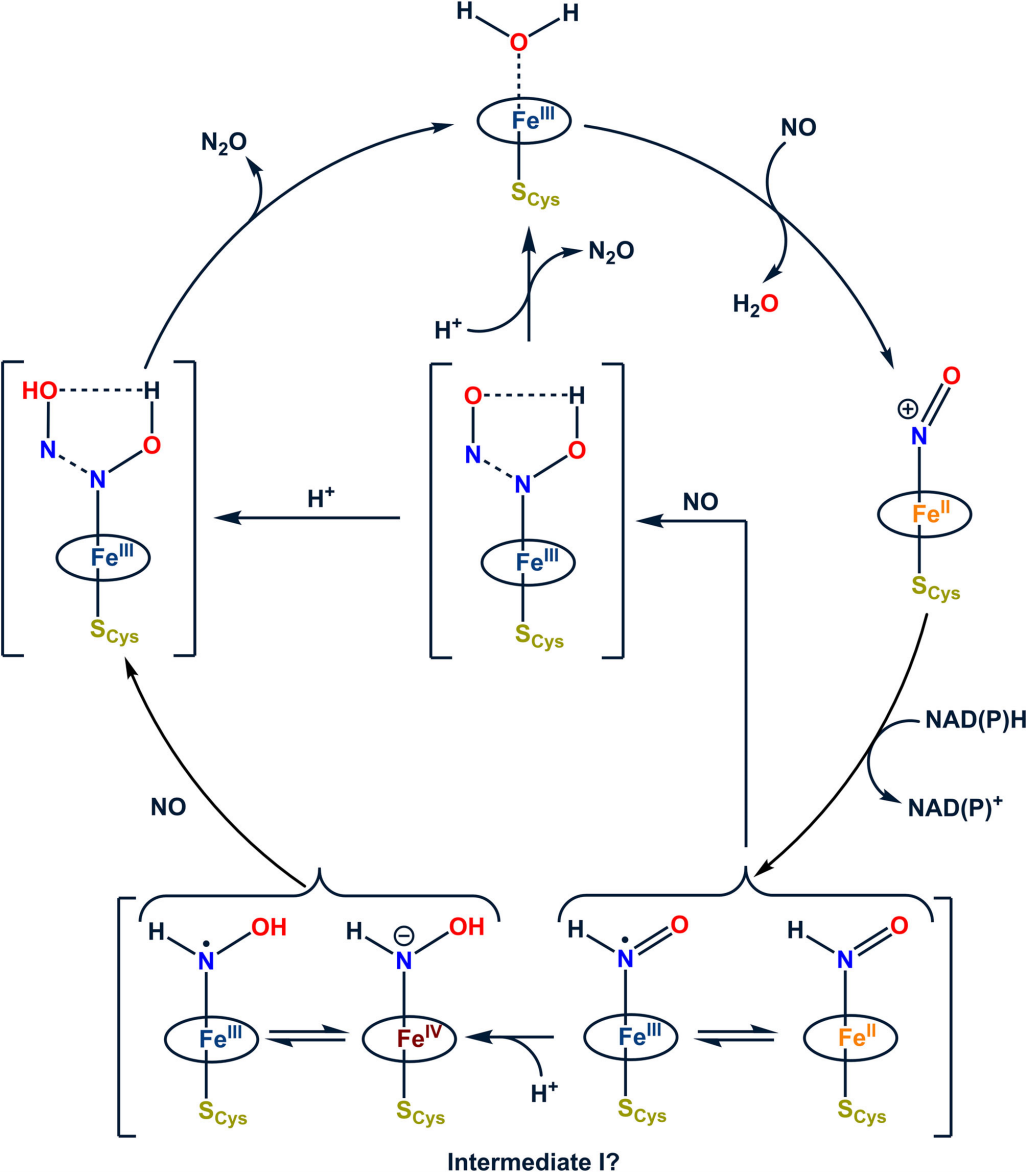
Proposed mechanism for Cyt. P450nor showing the possible hyponitrite intermediate(s) in the singly versus doubly protonated state.

The mechanism of NO reduction by Cyt. P450nor is still debated and under investigation but a proposed mechanism is shown in Scheme 1. In the first step, the ferric resting state reacts with NO to form a low‐spin (ls) {FeNO}^6^ species according to the Enemark‐Feltham notation. As NO is a noninnocent ligand, the Enemark‐Feltham notation is often used as an electron counting system here to describe the electronic structures of transition metal‐nitrosyl species [20]. The notation, written as {M(NO)_x_}^n^, counts the total number of valence electrons in the metal nitrosyl group by treating the metal‐nitrosyl core as a single covalent unit, where x is the total number of NO ligands, and the number n is calculated by adding the d electrons from the metal and the π^*^ electrons from the NO ligand(s). Over the years, the Enemark‐Feltham notation has been expanded to include the spin state of the metal in the MNO unit [1]. The ls‐{FeNO}^6^ adduct of Cyt. P450nor has been characterized by UV‐Vis, IR, and rRaman spectroscopy along with X‐ray crystallography (see Figure 1A) [18, 21, 22, 23]. The ls‐{FeNO}^6^ adducts can formally be described as Fe(II)‐NO^+^ species with a strong Fe(II)‐to‐NO^+^ π‐backbond and an electrophilic NO^δ+^ ligand (due to the strong π‐backbond, the formally positive charge of the NO ligand is largely diminished, leaving only a fractional positive charge on the NO ligand) [24, 25, 26]. Accordingly, the mechanism proceeds with NADH acting as a hydride donor to the ls‐{FeNO}^6^ species to form “Intermediate I” [21, 27, 28, 29]. This is a very short lived intermediate with a lifetime of approximately 100 ms, which has complicated the structural and electronic characterization of this species [1, 21]. As Intermediate I is generated through a hydride transfer to the ls‐{FeNO}^6^ species, it is rational to postulate that this species should resemble a ls‐{FeHNO}^8^ complex. Intriguingly, based on computational analysis, it has been proposed that the ls‐{FeNO}^8^ complex should be basic enough due to the cysteinate ligand to become protonated, which would generate an Fe–NHOH type species [30]. Detailed QM/MM studies of Cyt. P450nor and model complex studies also support the second protonation of Intermediate I with a proposed electronic structure of Fe^III^–NHOH^•^ [31, 32, 33]. Intermediate I has been characterized by UV‐Vis [21] and rRaman [34] spectroscopy; however, due to the short lifetime of this intermediate, further characterization of this species has, until recently, remained elusive. Nomura et al. recently used time‐resolved infrared (IR) spectroscopy, time‐resolved freeze trapped crystallography with an X‐ray free electron laser (XFEL), and QM/MM calculations to study the reaction of Cyt. P450nor with NO [35]. The authors reported, for the first time, the N−O stretching frequencies of Intermediate I at 1330 cm^−1^ and 1290 cm^−1^, which correspond to Intermediate I with and without NAD^+^ present in the protein's active site, respectively. This work suggests that Intermediate I is a singly protonated species, which the authors postulate to have an Fe^III^−NHO^•−^ electronic structure based on QM/MM results. The role of the axial thiolate (cysteinate) ligand in Cyt. P450nor would then be to enable the formation of the ferrous HNO complex in the alternative Fe^III^−NHO^•−^ valence tautomer, to enhance reactivity with NO in the next step of the reaction [35, 36].

In the final step of the mechanism, a second molecule of NO and H^+^ react with Intermediate I to eventually generate N_2_O and H_2_O; however, no intermediates of this process have thus far been experimentally identified. Based on chemical intuition and computational results, the second NO molecule should react with Intermediate I to form a new N–N bond, thus generating a formal hyponitrite intermediate [1, 30, 31, 32]. This hyponitrite unit would then be expected to tautomerize in order to be properly oriented to cleave the N–O bond to release N_2_O and generate H_2_O to close the catalytic cycle. In this case, the hyponitrite intermediate would be expected to be in the *cis* orientation (Scheme 1). Whether the hyponitrite unit is singly protonated or doubly protonated before N–O bond cleavage occurs is currently unknown and difficult to discern. QM/MM modeling indicates that there is little difference in energy between the singly protonated and doubly protonated forms of the hyponitrite intermediate [31]. A possible hint at the protonation state of the hyponitrite intermediate comes from synthetic model complexes. Recently, Manickas et al. prepared and studied a heme‐HNO model complex of Intermediate I [36]. In this case, upon addition of NO gas to the formed Fe‐HNO species, quantitative amounts of N_2_O were generated. This indicates that the second protonation may not be necessary in Cyt. P450nor to generate N_2_O and that the fleeting proposed hyponitrite intermediate that is formed may be in the singly protonated form. Further investigations using both synthetic model complexes and time‐resolved spectroscopic techniques on the Cyt. P450nor enzyme may help elucidate the protonation state of the key hyponitrite intermediate formed after the N–N coupling step, on the road to N_2_O formation.

### 2.2 Cytochrome *c* Nitric Oxide Reductase (cNOR)

In contrast to fungal NORs, bacterial NORs use a completely different active site and mechanism to catalytically reduce NO to N_2_O. Denitrifying bacteria contain a periplasmic membrane‐bound enzyme known as *c*NOR (or NorBC) that is capable of the two‐electron reduction of NO [1, 37]. These bacterial NORs belong to the superfamily of heme‐copper oxidases, with the most prominent members of this family being Cyt. *c* oxidases (C*c*Os), which are responsible for the energy generation in aerobic respiration [38, 39]. There are several types of bacterial NORs that are distinguished by their electron sources and electron transfer centers, as reviewed recently [1]. The structures of bacterial NORs have primarily been investigated for the two‐subunit enzyme *c*NOR, which has been largely purified from organisms of the *Pseudomonas* family [40]. These enzymes are heterodimers with two subunits: NorB and NorC. NorC is responsible for electron transfer to the NorB subunit, which contains the catalytically active site for NO reduction. Here, we only focus on the *c*NOR active site in relation to the key hyponitrite chemistry, and therefore point the reader elsewhere for a more complete picture of the structure of the *c*NOR heterodimer [1]. The active site of *c*NOR contains two distinct iron sites, a non‐heme iron center labeled Fe_B_ and a heme *b*_3_ center [4]. It is well known that membrane proteins are generally difficult to express, purify, and crystallize and *c*NOR is not an exception. The first crystal structure of *c*NOR was reported in 2010 by Shiro and coworkers at a resolution of 2.7 Å (PDB: 3O0R) [19] as shown in Figure 1B. The non‐heme Fe_B_ center is coordinated by three histidine residues and a glutamate whereas the heme *b*_3_ center is bound by an axial histidine residue. In the resting state, the Fe centers are bridged by an oxo ligand putting the Fe centers at an approximate distance of 3.8 Å.

The mechanism of how *c*NORs reduce NO remains highly debated and under investigation. It is now rather established that the resting state of *c*NOR is fully oxidized with a ferric heme and a ferric non‐heme iron site and a bridging μ‐oxo group, as has been confirmed by UV‐Vis, rRaman, MCD spectroscopy, and X‐ray crystallography [19, 37, 41]. In the presence of the μ‐oxo group, the axial histidine residue is not bound at the heme center, based on rRaman data, which, however, contrasts with the crystal structure of *c*NOR (Figure 1B) [19, 37, 41]. This deviation may be a result of crystal packing or a lack of enzyme dynamics in the solid state, but further work is needed to investigate this point. Upon one‐electron reduction of the *c*NOR active site, the μ‐oxo linkage is broken, leading to the rebinding of the axial histidine to the heme *b*_3_ center. The distal ligand bound at the ferric heme is either water or hydroxide; however, spectroscopic and crystallographic data are lacking for a definitive assignment. The dependence on pH was investigated as well, and those results revealed that the distal ligand has a strong role in modulating the redox potential of the heme. Increasing reactivity at lower pH was further observed, which suggests that the distal ligand is more likely to be water than hydroxide [37, 42, 43, 44]. The second electron transfer then generates the fully reduced diferrous enzyme. In this state, the active site is proposed to contain a six‐coordinate heme *b*_3_ with an axial histidine residue and a bound water ligand [42, 43]. Upon the addition of NO to fully reduced *Psudomonas aeruginosa c*NOR under single turnover conditions, a five‐coordinate ferrous heme‐nitrosyl adduct forms as observed by EPR spectroscopy [45, 46]. Under catalytic turnover conditions, on the other hand, accumulation of a non‐heme hs‐{FeNO}^7^ adduct was also observed [47]. These findings, however, are difficult to rationalize as the actual on pathway intermediate with NO bound to both the heme and the non‐heme iron site would likely be a spin‐coupled system, which should be EPR silent [48]. Recently, a time‐resolved UV‐Vis and IR spectroscopic study indicates three initial mechanistic stages [49]. In the first stage, NO enters the active site and binds to the non‐heme iron center, forming a hs‐{FeNO}^7^ adduct on the microsecond timescale. In the second stage, NO migrates to form a possibly five‐coordinate heme‐NO complex which occurs again on the microsecond timescale. Finally, a second molecule of NO enters the active site on the millisecond timescale, leading to electron transfer and NO coupling to ultimately form N_2_O. Unfortunately, a complete experimental picture of the NO coupling mechanism of *c*NORs is still lacking, hampered by the difficulty of working with these membrane‐bound proteins.

**SCHEME 2:**
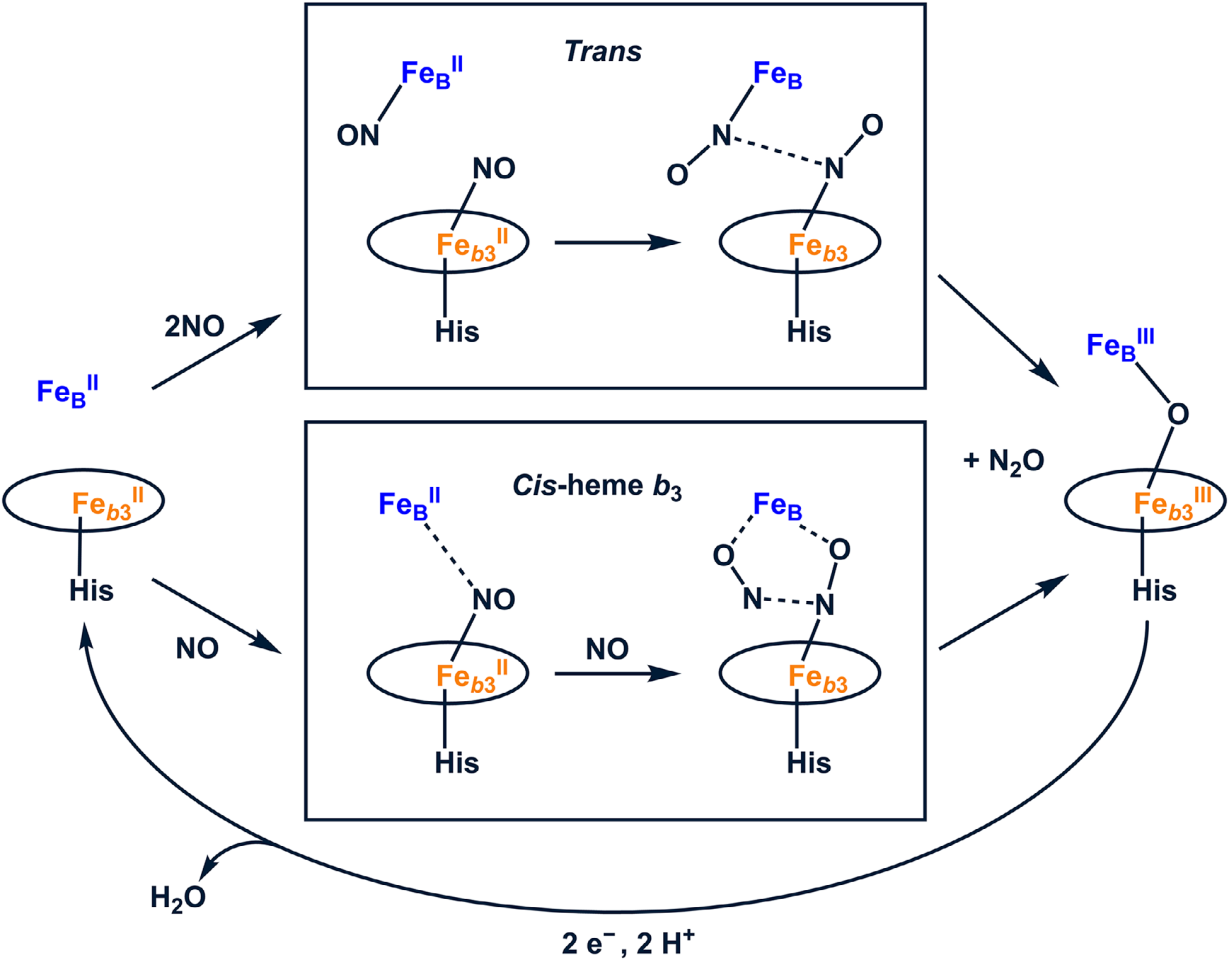
Proposed mechanism for NO reduction by *c*NOR.

Based on the current data on *c*NORs and our knowledge of iron‐nitrosyl chemistry in general [1], three major mechanisms have been proposed for these enzymes [7]. Of these mechanisms, only the *trans* mechanism and the *cis*‐heme *b_3_* mechanism are still under consideration. Importantly, both mechanisms are speculative as so far, the key intermediate prior to N–N bond formation has not been observed experimentally for any *c*NOR. For the *trans* mechanism, it has been proposed that a molecule of NO binds at both the non‐heme Fe_B_ and the heme *b*_3_ site. Radical‐radical N–N coupling is proposed to occur from this dinitrosyl intermediate to form a bridging *trans*‐hyponitrite intermediate (see Scheme 2), before the generation of N_2_O and the formation of a bridging oxo group between the Fe centers [50]. The proposed *trans‐*hyponitrite intermediate contrasts with the *cis*‐hyponitrite intermediate expected for Cyt. P450nor. Experimental support for this *trans* mechanism has specifically come from the observation of NO binding at both the non‐heme Fe_B_ and heme *b*_3_ site during catalysis as mentioned above, and a protein model for the enzyme [47, 51]. Unfortunately, no *trans*‐hyponitrite intermediate has been experimentally observed to date [1]. Furthermore, from a fundamental perspective, both heme and non‐heme {FeNO}^7^ species tend to be quite stable and unreactive [52, 53]. Computational studies by Blomberg and coworkers on the mechanism of *c*NORs predict that N–N coupling from the *trans*‐hyponitrite intermediate would be too high in energy to be feasible, which indicates the operating pathway in *c*NORs may not in fact be the *trans* mechanism [54, 55]. Further work is needed to rule out the *trans* mechanism through further spectroscopic studies on *c*NORs and complementary studies on synthetic and protein model systems.

An alternative mechanism for NO reduction by *c*NORs is the *cis*‐heme *b*_3_ mechanism (see Scheme 2). This mechanism was strongly inspired by the findings in Cyt. P450nor enzymes as discussed above [1]. In this route, the heme Fe‐NO adduct forms first and is then subsequently attacked by a second NO molecule. Based on computational results it is suggested that the Fe_B_ center acts as a Lewis‐acid mediator, polarizing the heme Fe‐NO unit, thereby priming it for coupling with a second NO molecule while simultaneously stabilizing the proposed hyponitrite intermediate [54, 55]. Some experimental support for this mechanism comes from flash photolysis experiments [56] and time‐resolved UV‐Vis and IR spectroscopy experiments [49]. Further experimental evidence would be needed to definitively distinguish between the *trans* and *cis*‐heme *b*_3_ mechanism. Interestingly, the proposed hyponitrite intermediates in these two pathways have different structures and would therefore have rather distinct vibrational modes from one another [57, 58]. Capturing these intermediates in *c*NORs or in synthetic model complexes is paramount to gain a further understanding of NO reduction in these enzymes.

### 2.3 Flavodiiron Nitric Oxide Reductases (FNORs)

In contrast to bacterial and fungal NOR enzymes that operate in the respiratory chain, FNORs are utilized by pathogenic bacteria to counteract the key mammalian immune defense agent NO [59]. These pathogens have evolved a strategy in which a transcription factor senses NO [60], thereby inducing transcription of FNORs to reduce NO to less toxic N_2_O [61]. FNORs belong to the family of Flavodiiron Proteins (FDPs), which are found within certain bacteria, archaea, and protozoa [8, 9, 62]. The primary coordination sphere of the non‐heme diiron active site in FDPs is generally conserved. In the diiron core of the enzymes from *T. maritima* and *M. thermoacetica*, each iron is five‐coordinate with two histidine residues and a monodentate carboxylate ligand (either an aspartate or a glutamate residue; Figure 1C). Additionally, the two iron centers are bridged by a hydroxide and a carboxylate ligand (usually an aspartate residue), which positions the iron centers between 3.3 and 3.6 Å apart [9, 63, 64, 65]. A notable exception to the conserved primary coordination sphere is *Desulfovibrio gigas* FDP, which shows a slightly more asymmetric diiron site. In this case, one of the histidine moieties is replaced by a water molecule [66]. Despite this difference in the primary coordination sphere ligation between typical FDPs and the *D. gigas* enzyme, this structural change does not seem to cause a difference in reactivity [64]. Furthermore, all available crystal structures of FDPs, regardless of primary ligand coordination, show one open coordination site per iron center, which allows two molecules of NO to bind in a *cis* orientation, which is important for facile N–N bond formation and N_2_O generation [1, 67].

**SCHEME 3:**
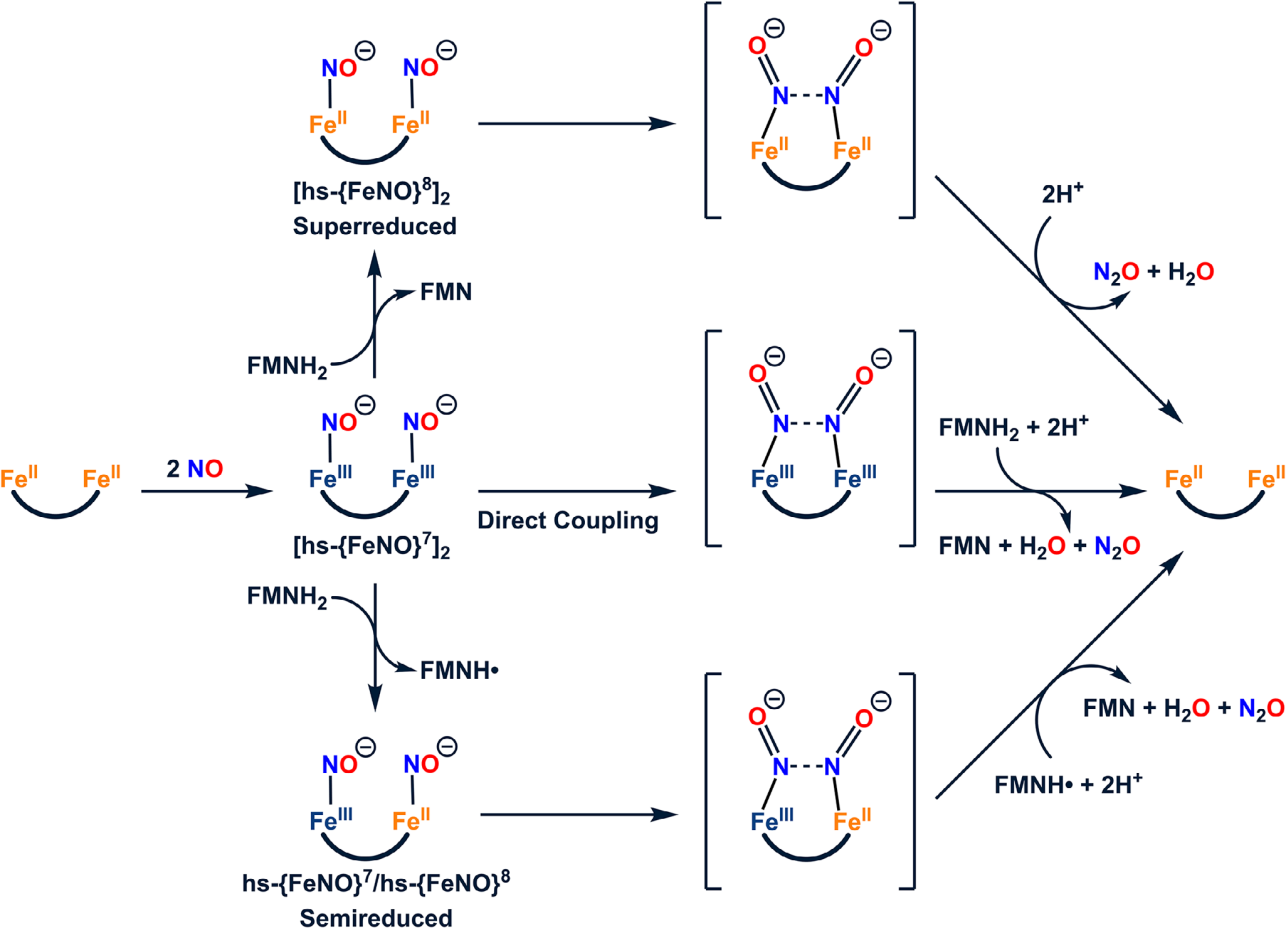
Proposed mechanism for NO reduction by FNORs showing possible Fe–hyponitrite intermediates.

Despite extensive studies on NO activation by FNORs using both enzymes and synthetic model complexes, the exact mechanism of NO reduction by these diiron enzymes is still contested [1]. Kurtz and coworkers have utilized rapid‐freeze quench Mössbauer‐ and EPR‐spectroscopic studies on the flavinated and deflavinated *T. Maritima* FDP to explore how N–N coupling occurs in this enzyme [68, 69, 70]. Their results reveal that although a diiron mononitrosyl hs‐Fe^II^/{FeNO}^7^ intermediate is generated in the enzyme when sequential additions of NO equivalents are made, the diiron dinitrosyl species [hs‐{FeNO}^7^]_2_ is the catalytically relevant intermediate that forms before any N_2_O generation [69, 70]. From here, three different pathways for N–N coupling are theoretically feasible and depend on the question as to how electrons are delivered from the FMN cofactor to the diiron core during the reaction. The three proposed pathways are known as the “direct reduction”, “semireduction”, and “superreduction” mechanisms (Scheme 3) [1]. In the “direct reduction” pathway, the [hs‐{FeNO}^7^]_2_ intermediate is proposed to generate N_2_O without the participation of the FMN cofactor, only requiring the two electrons from the cofactor to regenerate the active site after the catalytic cycle completes [69, 70, 71, 72]. Alternatively, the “semireduction” pathway would require one electron equivalent from the FMN cofactor to activate the [hs‐{FeNO}^7^]_2_ dimer, forming a hs‐{FeNO}^7^/hs‐{FeNO}^8^ intermediate prior to N_2_O formation [67, 73, 74, 75]. Finally, the “superreduction” pathway has been proposed to require two electron equivalents from the FMN cofactor to form a [hs‐{FeNO}^8^]_2_ intermediate prior to N_2_O generation.

Even with extensive experimental efforts, intermediates of NO reduction by FNORs, such as the elusive hyponitrite intermediate(s), have yet to be observed [1]. Computational studies indicate that N–N coupling would occur in a coplanar fashion, likely forming a *cis*‐hyponitrite intermediate [76, 77]. We have demonstrated experimentally that a coplanar orientation of the two NO ligands is in fact a requirement for facile N–N coupling to occur, directly supporting this notion [67]. We were unable to observe any evidence for the hyponitrite intermediate in our model systems either, even in low‐temperature studies, with only the formation of N_2_O detected upon NO reduction [67, 72, 74]. Another interesting observation is that the oxidation state of the metal center will be different for the Fe‐hyponitrite intermediate depending on the mechanistic pathway. Here, formally Fe^II^Fe^II^‐, Fe^II^Fe^III^‐, or Fe^III^Fe^III^‐hyponitrite species would be expected to form as the intermediates upon N–N coupling (Scheme 3). These species would not only possess drastically different spectroscopic features but one would expect that the stability of the formed Fe‐hyponitrite species would change based on the oxidation state of the metal centers [78]. The Fe oxidation state and hyponitrite reactivity are discussed in more detail in the synthetic model complex section below.

In support of the “direct reduction” pathway, Kurtz and coworkers have demonstrated that even in the absence of the FMN cofactor, the *Tm* FDP is capable of a single turnover of 2 NO molecules to N_2_O from the diferrous resting state, but note that this occurs at a slower rate than the reaction in the flavinated FDP [69]. In this case, both reductive equivalents needed for NO reduction originate from the diferrous core in a “direct reduction” mechanism. This implies that the FMN cofactor only acts as a means of re‐reducing the active site after N–N coupling to the diferrous active state. This is rather surprising based on the numerous examples of hs‐{FeNO}^7^ model complexes [67, 73, 74, 79, 80, 81] in the literature that are stable toward N–N coupling due to the high covalency of the Fe–NO bond [1, 82]. Recently, however, computational studies have identified a second coordination sphere (SCS) tyrosine residue within hydrogen bonding distance that may contribute toward the ability of FNORs to follow the direct reduction pathway [71, 76, 83]. Chen and coworkers have proposed that the conserved Y197 residue in FNORs (residue 197 in *Tm* FDP; Figure 1C) is able to hydrogen bond to one of the coordinated NO ligands, leading to a decrease in both the enthalpic and entropic penalties toward N–N bond formation and subsequent N–O bond cleavage in the NO reduction pathway [84]. In other words, protonation may be required to form the subsequent hyponitrite intermediate. The computational results support the formation of *cis‐*hyponitrite intermediate(s) during the N–N coupling stage of NO reduction, despite the lack of experimental evidence for these elusive intermediates. Site‐directed mutagenesis studies have demonstrated that the Y197 residue is crucial for NO reduction activity, supporting the computational results [71]. Quite recently, we probed the role of the SCS in NO reduction at non‐heme iron centers using synthetic model complexes [85]. We demonstrated that the incorporation of strong SCS H‐bond donors allows for the direct reduction of NO to N_2_O. Taken together, these results strongly imply that the key intermediate in NO reduction by FNORs is a protonated hyponitrite species.

## 3 Synthetic Iron‐Hyponitrite Complexes

The lack of any observable Fe‐hyponitrite intermediate(s) in NORs is one of the major gaps in our mechanistic understanding as to how Nature reduces NO to N_2_O. Our group, and many others, have employed synthetic model complexes and protein models as an alternative approach to gain mechanistic insights into NOR metalloenzymes and potential reaction pathways [25, 29, 33, 36, 67, 72, 73, 74, 75, 84, 85, 86, 87, 88, 89, 90, 91, 92, 93, 94, 95, 96, 97, 98, 99, 100]. Advantages of using synthetic model complexes are that one can more easily tune the first coordination sphere of the metal center, control the addition of protons and electrons, and work in organic solvents that are more amenable to low‐temperature (−80 °C) reactivity studies for trapping intermediates. One of the major disadvantages of synthetic model complexes is that the ability to tune the environment outside of the first coordination sphere is quite limited. Despite this limitation, synthetic model complexes have been successfully used to provide insights into the nature and stability of reaction intermediates, alternative reaction pathways, and SCS tuning of the reactivity for NO reduction in NOR enzymes [1, 67, 81, 85, 100]. Although a significant number of synthetic Fe‐nitrosyl complexes have been reported, the number of structurally characterized Fe‐hyponitrite complexes remains quite limited. There are only three reported Fe‐hyponitrite complexes to date [78, 101, 102] and they are described in detail below.

But before we get there, one cannot help but wonder why there are only three reported Fe‐hyponitrite complexes in the literature, but hundreds of Fe‐nitrosyl species? The obvious answer is that Fe‐nitrosyl complexes are much more stable than Fe‐hyponitrite complexes. Another major issue is the limitation of the current synthetic toolbox to prepare Fe‐hyponitrite species. There are two main synthetic strategies available to access Fe‐hyponitrite complexes. The first involves the reactivity of an Fe complex (ferrous for non‐heme iron and ferrous or ferric for heme iron) with NO gas. This method has been successful for preparing other metal‐hyponitrite complexes [11], but has been largely unsuccessful for iron. It has been shown for electron‐rich non‐heme diiron complexes that direct N–N coupling can occur, analogous to the mechanism of FNORs, but in these cases Fe‐hyponitrite intermediate(s) could not be trapped either, even at low‐temperatures [72, 100]. With less electron rich diiron centers, a one‐electron reduction of the stable dinitrosyl adduct is necessary to facilitate N–N coupling, but this again leads to the formation of N_2_O without the detection of Fe‐hyponitrite intermediate(s), even at low temperature [67, 74]. The only successful preparation of a Fe‐hyponitrite complex from NO gas directly was reported for a dinitrosyl iron complex (DNIC) and is described in more detail below [102]. The alternative method is using a source of pre‐formed hyponitrite. This has been a successful method for forming metal‐hyponitrite species [11] in general, and importantly, for forming Fe‐hyponitrite complexes [78, 101]. Using pre‐formed hyponitrite (in the form of the salt or acid) to form Fe‐hyponitrite species is advantageous, as the exact stoichiometric addition of hyponitrite can be more easily conducted compared to NO gas. Furthermore, the binding of pre‐formed hyponitrite with an Fe center is a redox neutral process compared to the one‐electron oxidation that occurs upon binding of NO gas, followed by N–N coupling to form hyponitrite. This former approach allows one to study the reaction of hyponitrite with Fe centers in different oxidation states, which is important for understanding the Fe‐hyponitrite intermediate(s) involved in N–N coupling in FNORs [78]. To design reactions of Fe complexes with pre‐formed hyponitrite, it is important to first provide a background into the properties of simple hyponitrite salts.

### 3.1 Hyponitrite Salts

Well before the discovery of NOR enzymes, chemists had already begun to investigate hyponitrite salts. The first reports of the formation of hyponitrite salts date back to the 19^th^ century [103, 104]. Since then, many forms of hyponitrite salts have been reported in the literature, but we will focus our attention here on two pre‐formed hyponitrite salts that are most relevant to reactions with transition‐metal complexes: Na_2_N_2_O_2_, Ag_2_N_2_O_2_, and the free acid H_2_N_2_O_2_. These species represent the most well characterized forms of hyponitrite and have been used successfully to prepare metal‐hyponitrite species reported in the literature [11], including two Fe‐hyponitrite complexes [78, 101], which are discussed in more detail below.

#### 3.1.1 Sodium *Trans*‐Hyponitrite

Hyponitrite can exist in two possible isomers: the *trans* and *cis* form [11]. Of these forms, *trans*‐hyponitrite is the most common and well characterized. Sodium *trans*‐hyponitrite can be prepared by reducing a cold (0 °C) aqueous solution of sodium nitrite with Na/Hg amalgam [105, 106, 107, 108]. Alternatively, sodium *trans*‐hyponitrite can be prepared directly from reacting alkyl nitrites with hydroxylamine in an ethanolic solution of sodium ethoxide [109, 110, 111]. Finally, sodium *trans*‐hyponitrite can be prepared directly from NO gas using sodium/benzophenone in a 1,2‐dimethoxyethane/toluene solution [112]. Each of these methods gives rather poor overall yields, and very little improvement or development in the synthetic preparation of sodium *trans*‐hyponitrite has been made since the early 20^th^ century. Sodium *trans*‐hyponitrite is now commercially available as the hydrated salt; however, for isotope labelling studies, it must still be synthesized directly from a ^15^N source via one of the methods described above. Based on convenience, Na_2_^15^N_2_O_2_ has been primarily prepared from reducing Na^15^NO_2_ with Na/Hg amalgam [78, 101].

Single‐crystal X‐Ray diffraction data of sodium *trans*‐hyponitrite as the pentahydrate salt have been reported [113]. The salt features an N–O bond distance of 1.3622(11) Å and an N–N bond distance of 1.256(2) Å, which is reminiscent of single bond and double bond character, respectively. Furthermore, the structure shows an N–N–O angle of 112.14(9)°. Finally, the Na–O contact distance was determined to be 2.5178(8) Å. This information is summarized in Table 1. Comparison of the structure of this salt with those of Fe‐hyponitrite complexes is provided below. In addition to structural information, sodium *trans*‐hyponitrite has also been investigated and characterized by vibrational spectroscopy. Throughout the years, there have been several vibrational studies on sodium *trans*‐hyponitrite with conflicting reports of the vibrational stretching frequencies of this simple salt [57, 114, 115, 116, 117, 118, 119]. For example, the antisymmetric N–O stretch of sodium *trans*‐hyponitrite has been reported between 1035 – 1015 cm^−1^ as measured by IR spectroscopy. A major complication in determining the vibrational modes and frequencies of sodium *trans*‐hyponitrite accurately is the fact that synthesized sodium *trans*‐hyponitrite is often contaminated with impurities such as Na_2_CO_3_. In addition, the number of hydrates may vary based on how sodium *trans*‐hyponitrite was dried. Nonetheless, the reported range for the antisymmetric N–O stretch of sodium *trans*‐hyponitrite has been quite valuable for assigning this vibrational feature in Fe‐hyponitrite complexes [78, 101, 102].

**TABLE 1:** Key bond parameters of different forms of hyponitrite (see text for references).

Compound	N–N bond, Å	N–O bond, Å	N–N–O angle, deg	Other parameters	
*trans*‐Na_2_N_2_O_2_•5H_2_O	1.256(2)	1.3622(11)	112.14(9)	Na–O	2.5178(8) Å
*trans*‐H_2_N_2_O_2_	1.226(4)	1.363(3)	109.9(3)	—	—
*cis*‐Na_2_N_2_O_2_ ^a^	1.20(3)	1.40(3) 1.39(3)	119.8(4) 119.2(4)	—	—
[(OEP)Fe]_2_(μ‐N_2_O_2_)	1.250(3)	1.375(2)	108.5(2)	Fe–O	1.889(2) Å
				Fe–N(por)	2.049(2) – 2.064(2) Å
				∠Fe–O–N	118.56(2)°
[Fe_2_(NO)_4_(μ‐bdmap)]_2_(κ^4^‐N_2_O_2_)	1.279(5)	1.330(3)	112.8(3)	Fe–O	2.034(2) Å
				Fe–N	2.143(3) Å
				∠Fe–N–O	122.01(19)°
				∠Fe–O–N	113.57(18)°
[{Fe_2_(BMPA‐PhO)_2_}_2_(μ‐N_2_O_2_)]^2+^	1.257(8)	1.358(6)	114.2(6), 113.4(6)	Fe–O	1.981(3) Å, 2.026(3) Å
				Fe–N	2.214(5) Å, 2.211(5) Å
				∠Fe–N–O	107.1(3)°, 103.1(3)°
				∠Fe–N–N	141.9(6)°, 138.0(6)°
				∠Fe–O–N	120.5(3)°, 123.4(3)°

^a^ *Note: cis*‐Na_2_N_2_O_2_ bond parameters were determined from powder XRD rather than conventional single‐crystal XRD analysis.

#### 3.1.2 Sodium *Cis*‐Hyponitrite

Compared to sodium *trans*‐hyponitrite, sodium *cis*‐hyponitrite is rather underexplored, likely due to its reported instability and the synthetic difficulties in obtaining this compound. It has been reported that sodium *cis*‐hyponitrite can be prepared by reducing NO gas in the presence of sodium metal in liquid ammonia [120]. This is surprising to us as a similar reaction in toluene (described above) produces sodium *trans*‐hyponitrite. Although it is possible that the polarity of the solvent and possible hydrogen bonds with ammonia play a role in the formation of *cis*‐hyponitrite in this reaction, we believe this synthetic preparation method should be re‐investigated. Alternatively, sodium *cis*‐hyponitrite can be prepared in a tube furnace by reacting N_2_O gas with Na_2_O at 360 °C in a sealed tube for 2 h [58]. The authors note that the reaction is highly sensitive to temperature, N_2_O pressure, and reaction time. The sensitivity of the reaction along with the requirement of specialized equipment make this preparation less than desirable for most synthetic research groups. Recently, it was reported that sodium *cis*‐hyponitrite can be prepared in a milder manner by ball milling Na_2_O in the presence of N_2_O gas [121]. Although this method still requires specialized equipment, the mild reaction conditions make this synthetic method more appealing. Thus far, single‐crystal X‐ray diffraction data have not been reported for sodium *cis*‐hyponitrite, likely due to the solid‐state reactions required to generate this salt. Nevertheless, powder X‐ray diffraction has provided some insights into the structure of sodium *cis*‐hyponitrite, indicating an N–N bond distance of 1.20(3) Å and an N–O bond distance around 1.40(3) Å (Table 1), which are similar to sodium *trans*‐hyponitrite. The N–N–O bond angle is around 119°, significantly larger than that of the *trans* isomer. The vibrational properties of sodium *cis*‐hyponitrite also vary from those of sodium *trans*‐hyponitrite [58]. For sodium *cis*‐hyponitrite, the reported N–O stretching frequencies are at higher energies: 1065 cm^−1^ and 1097 cm^−1^. In theory, this provides a good handle for identifying *cis*‐ versus *trans*‐hyponitrite species in metal complexes. We note though that in Fe‐hyponitrite complexes the Fe center has a strong influence on the energy of the antisymmetric N–O stretching mode [78, 101, 102] (discussed further below), and that a vibrational frequency by itself is not enough to determine the structure of the bound hyponitrite and distinguish the two isomers. DFT calculations predict that the *cis*‐hyponitrite isomer is more stable than the *trans*‐hyponitrite isomer by approximately 1.6 kJ/mol [122]. This is surprising, as sodium *cis*‐hyponitrite appears to be more difficult to prepare than the *trans*‐hyponitrite analog. It should be noted, however, that 1.6 kJ/mol is within the expected error in DFT‐predicted total energies, which is approximately ±5 kcal/mol (about ±21 kJ/mol). Additionally, these DFT calculations were conducted by calculating the energies for the free hyponitrite isomers in the gas phase, absent of the sodium cations, which may influence the predicted stability of the corresponding sodium hyponitrite salts.

#### 3.1.3 Silver *Trans*‐Hyponitrite

Silver hyponitrite can be prepared by reacting an aqueous solution of sodium *trans*‐hyponitrite with AgNO_3_ [108, 109, 112, 123]. This method is convenient, as Ag_2_N_2_O_2_ directly precipitates from solution. As silver hyponitrite is prepared from the *trans*‐hyponitrite salt, silver *trans*‐hyponitrite is expected to form, but structural confirmation through single‐crystal X‐ray diffraction has not yet been reported for this salt. Compared to sodium *trans*‐hyponitrite, the vibrational modes of silver hyponitrite are shifted to slightly higher energies [57]. Here, the antisymmetric N–O stretching frequency for silver hyponitrite is reported at 1058 cm^−1^, 23 cm^−1^ higher in energy than that of sodium *trans*‐hyponitrite reported in the same manuscript. This shift is likely due to the difference between the sodium and silver cations rather than a structural change of the hyponitrite dianion. Silver hyponitrite has been used to prepare a Cu‐hyponitrite complex [124] but thus far has not been used for Fe‐hyponitrite chemistry. One of the major drawbacks of silver hyponitrite for Fe‐hyponitrite chemistry is that silver(I) is a known oxidant [125] and instead of facilitating ligand exchange, may instead oxidize a ferrous iron to the ferric state. Silver hyponitrite may be useful for preparing ferric‐hyponitrite complexes (such as heme‐hyponitrite species) via ligand exchange, but this method has not been employed to date for this purpose.

#### 3.1.4 Hyponitrous Acid

Hyponitrous acid (HONNOH) can be prepared directly from silver hyponitrite by adding anhydrous HCl in diethyl ether [108, 113, 126]. In this case, only the *trans*‐hyponitrous acid is expected to form [11]. Hyponitrous acid can be isolated as a white crystalline solid; however, this is not recommended as this compound has been reported to be explosive in the solid state [113]. Instead, it is safer to prepare hyponitrous acid in situ and use it directly without isolation. Single‐crystal X‐ray crystallography revealed the cocrystallization of the acid as the salt [HEt_2_NCH_2_CH_2_NEt_2_H][HN_2_O_2_]•H_2_N_2_O_2_ [113]. In this structure, hyponitrous acid has a slightly shorter N–N bond distance of 1.226(4) Å compared to sodium *trans*‐hyponitrite and a similar N–O bond distance of 1.363(3) Å. The N–N–O angle is also slightly smaller than that of sodium *trans*‐hyponitrite at 109.9(3)°. The vibrational data for hyponitrous acid show two vibrational bands at 1014 cm^−1^ and 1004 cm^−1^ for the antisymmetric N–O stretching frequency [117], which is slightly lower in energy than the reported stretching frequencies for sodium and silver hyponitrite. Hyponitrous acid presents a very useful form of hyponitrite for preparing Fe‐hyponitrite complexes as unlike its sodium and silver counterparts, it is soluble in organic solvents. Hyponitrous acid has already been used to prepare a Fe‐hyponitrite complex [101], which is further discussed below.

#### 3.1.5 Other Hyponitrite Salts/Transfer Reagents

In addition to the forms of hyponitrite discussed above, some other forms of hyponitrite have been prepared. For example, several ammonium and base‐stabilized hyponitrite salts have been prepared and characterized [113]. The advantage of these organic salts compared to the sodium‐ and silver‐hyponitrite analogs is that they have higher solubility in organic solvents. Unfortunately, several of these salts are reported as unstable and potentially explosive. What governs the stability of some of these organic hyponitrite salts as opposed to others remains unknown and underexplored. Recently, stannyl‐hyponitrite species have been reported, which also appear to be highly soluble in organic solvents [127, 128]. In the presence of an acid, transmetallation of Sn with a Ru complex was reported to form a Ru‐hyponitrite complex [127]. Whether this method can be generally applied to other complexes and metals is unknown, but this may present an alternative method for forming Fe‐hyponitrite species. Finally, one other approach that has been reported, but remains underexplored overall, is the transfer of hyponitrite to a metal center from a diazeniumdiolate compound [129]. This Umpolung approach is quite unique as it requires the cleavage of a C–N bond to form the metal‐hyponitrite species. Importantly, this allows for the transfer of a *cis*‐hyponitrite ligand, a rare moiety due to the difficulty of preparing *cis*‐hyponitrite reagents (see above). We believe further exploration into the generality of this approach, along with the development of other diazeniumdiolate compounds capable of this chemistry, could greatly advance the field of metal‐hyponitrite chemistry and may open the door for preparing Fe‐hyponitrite complexes with the *cis* form of hyponitrite.

#### 3.1.6 Coordination of Hyponitrite to [Fe(OEP)]^+^

**SCHEME 4:**
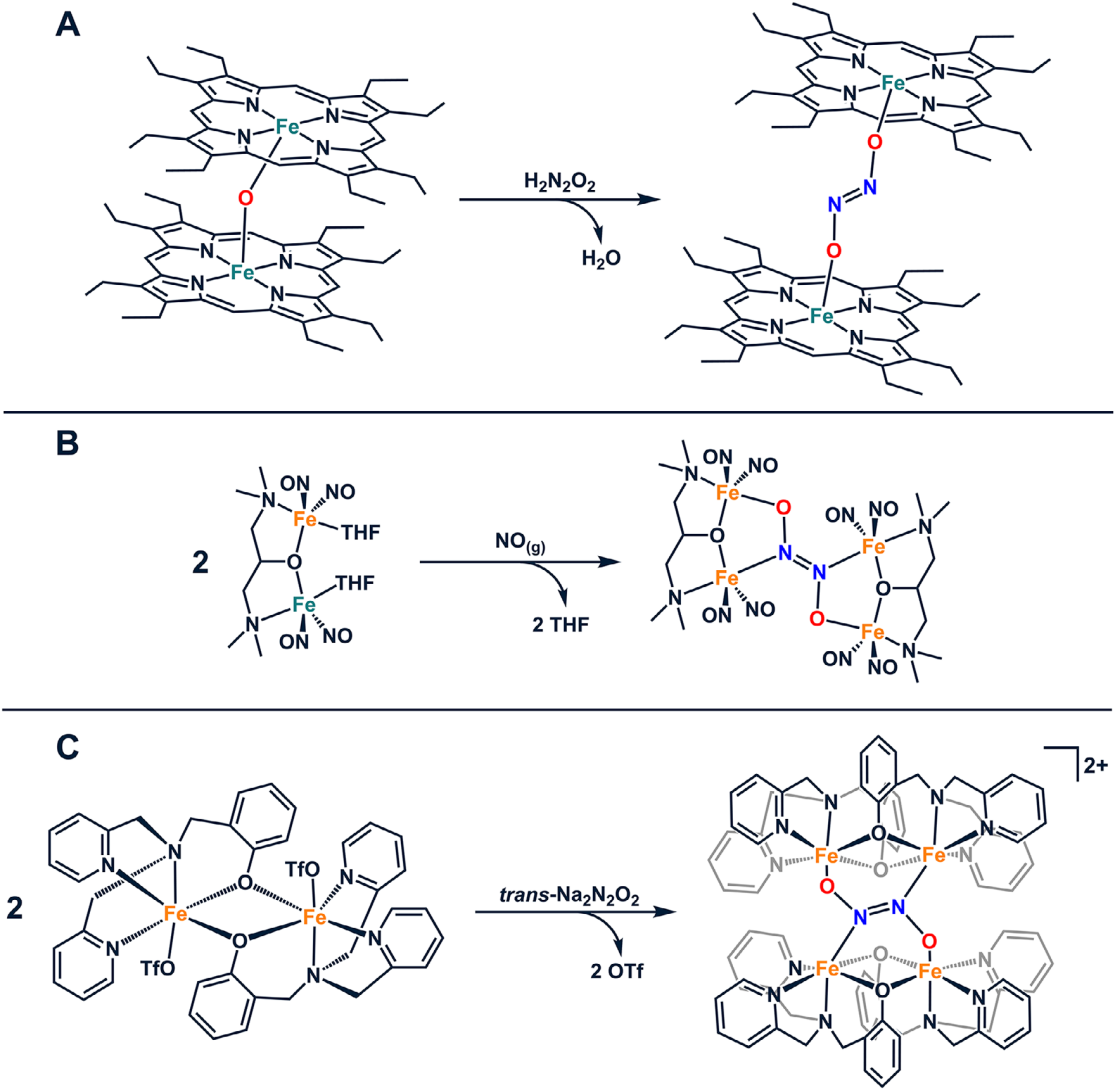
Synthetic routes used to prepare the three known Fe‐hyponitrite complexes. The synthesis of [Fe^III^(OEP)]_2_(μ‐N_2_O_2_) is shown in panel A, the synthesis of [Fe_2_(NO)_4_(μ‐bdmap)]_2_(κ^4^‐N_2_O_2_) in panel B, and the synthesis of [{Fe^II^_2_(BMPA‐PhO)_2_}_2_(μ‐N_2_O_2_)]^2+^ in panel C.

**FIGURE 2:**
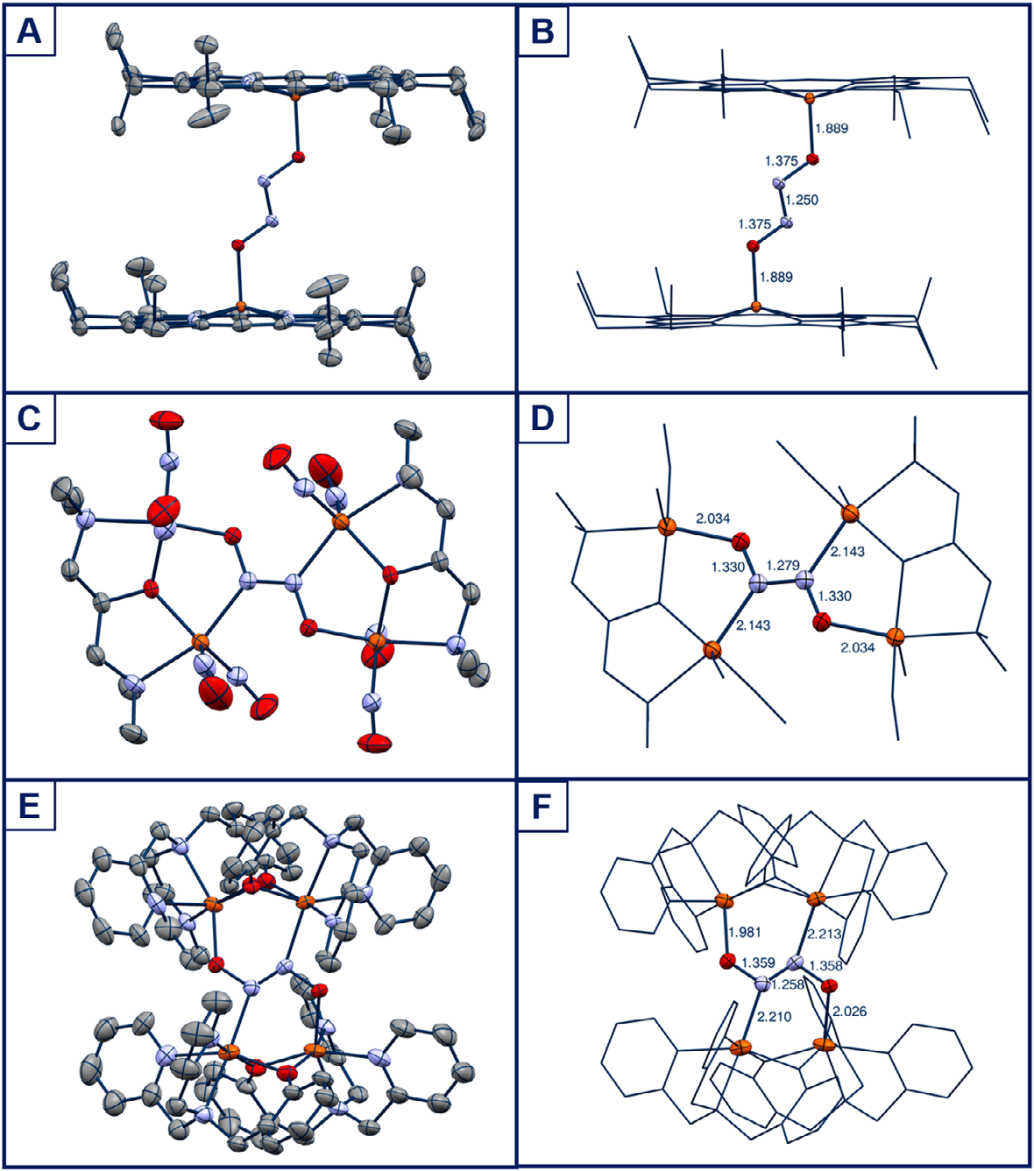
Different representations of each of the three reported Fe‐hyponitrite complexes. The left panels show the crystal structures of the Fe‐hyponitrite complexes. Fe atoms are orange, oxygen atoms are red, nitrogen atoms are light blue, and carbon atoms are gray, with ORTEPs drawn at 50% probability. Hydrogen atoms, solvent molecules, and outer sphere counterions have been omitted from all crystal structures for clarity. The right panels are a different representation of the crystal structures of the complexes with a focus on the Fe‐hyponitrite bonds. The representations of [Fe^III^(OEP)]_2_(μ‐N_2_O_2_) are shown in panels A and B. The representations of [Fe_2_(NO)_4_(μ‐bdmap)]_2_(κ^4^‐N_2_O_2_) are shown in panels C and D. The representations of [{Fe^II^_2_(BMPA‐PhO)_2_}_2_(μ‐N_2_O_2_)]^2+^ are shown in panels E and F.

As mentioned above, the full mechanistic picture of how *c*NOR is able to mediate NO reduction to N_2_O is still unclear [1]. Identifying whether *c*NOR operates through the *trans* or *cis*‐heme *b*_3_ mechanism would go a long way to providing a better mechanistic picture. Due to the difficulty of studying *c*NOR, synthetic model complexes may be a useful way to obtain further insight into the relevant chemistry. To help answer questions surrounding *c*NOR, Richter‐Addo and coworkers prepared the first dinuclear Fe‐hyponitrite complex (Scheme 4A) [101]. Here, [Fe^III^(OEP)]_2_(μ‐O) (OEP = octaethylporphyrin dianion) was reacted with anhydrous hyponitrous acid in toluene to produce [Fe^III^(OEP)]_2_(μ‐N_2_O_2_). This complex was structurally characterized by single‐crystal X‐ray diffraction, revealing a *trans*‐hyponitrite ligand bound at each of the negatively charged oxygen atoms, bridging between two Fe^III^(OEP) units (Figure 2). The N–N bond distance of the hyponitrite fragment was determined to be 1.250(3) Å, which is slightly elongated compared to the N–N bond distance of hyponitrous acid, but quite similar to that of sodium *trans*‐hyponitrite [113]. The N–O bond distance of the hyponitrite ligand of [Fe^III^(OEP)]_2_(μ‐N_2_O_2_) is 1.375(2) Å, which is elongated compared to the corresponding acid or sodium salt [113]. IR spectroscopy of [Fe^III^(OEP)]_2_(μ‐N_2_O_2_) revealed the antisymmetric N–O stretch of hyponitrite at 982 cm^−1^, which shifts to 973 cm^−1^ when the compound is prepared from H_2_^15^N_2_O_2_. This vibrational frequency is significantly lower in energy than that of hyponitrous acid, which indicates a weakening of the N–O bonds in the hyponitrite complex. Protonation of [Fe^III^(OEP)]_2_(μ‐N_2_O_2_) with HCl leads to the formation of N_2_O gas. We note that this reactivity is not unique and is now known to be common for metal‐hyponitrite complexes and for sodium *trans*‐hyponitrite in general [11, 78, 92, 102, 124, 130, 131, 132]. Later, [Fe^III^(OEP)]_2_(μ‐N_2_O_2_) was investigated using various spectroscopic techniques [53]. It was revealed that the Fe centers are best described as intermediate spin *S* = 3/2 species based on EPR and MCD spectroscopy, and SQUID magnetometry data. Here, very weak magnetic coupling occurs between the two Fe centers across the bridging *trans*‐hyponitrite ligand. Interestingly, it was observed that [Fe^III^(OEP)]_2_(μ‐N_2_O_2_) thermally decomposes over time to form 2 equivalents of the ls‐{FeNO}^7^ complex [Fe(OEP)(NO)], which is in essence the reverse reaction expected for *c*NOR (N–N bond cleavage of hyponitrite instead of N–N bond formation). This is likely due to the high thermodynamic stability of ls‐{FeNO}^7^ complexes, which is a known property of these kinds of compounds [1]. Overall, this result indicates that dimerization of two ferrous heme nitrosyls to form hyponitrite is thermodynamically unfavorable and suggests that the proposed *trans* mechanism for *c*NOR is unlikely [7]. However, we note that *c*NOR has a heme/non‐heme diiron active site whereas [Fe^III^(OEP)]_2_(μ‐N_2_O_2_) is a di‐heme model system. Investigation of a heme/non‐heme diiron model complex with pre‐formed hyponitrite could therefore provide more insight into this matter. Furthermore, the investigation of *cis*‐hyponitrite might shed light on the feasibility of the proposed *cis*‐heme *b*_3_ mechanism of NO reduction by *c*NOR [7]. To date, [Fe^III^(OEP)]_2_(μ‐N_2_O_2_) represents the only structurally characterized heme‐based Fe‐hyponitrite complex [101].

#### 3.1.7 The Hyponitrite Adduct of [(NO)_2_Fe(μ‐bdmap)Fe(NO)_2_(THF)]

As discussed above, the proposed Fe‐hyponitrite intermediate(s) for NO reduction in FNORs have not been observed in FNOR enzymes. Using synthetic model complexes, it may be possible to gain structural and/or electronic information regarding these key Fe‐hyponitrite intermediate(s). The first step toward this goal was reported by Liaw and coworkers [102]. Here, reaction of the complex [(NO)_2_Fe(μ‐bdmap)Fe(NO)_2_(THF)] (bdmap = 1,3‐bis(dimethylamino)‐2‐propanolate) with NO gas leads to the formation of the complex [Fe_2_(NO)_4_(μ‐bdmap)]_2_(κ^4^‐N_2_O_2_), representing the first Fe‐hyponitrite complex formed from NO gas rather than pre‐formed hyponitrite (Scheme 4B). [Fe_2_(NO)_4_(μ‐bdmap)]_2_(κ^4^‐N_2_O_2_) was structurally characterized by single‐crystal X‐ray diffraction (Figure 2) and shows a *trans*‐hyponitrite ligand bound to four Fe centers. The N–N and N–O bond distances for the hyponitrite ligand were found to be 1.279(5) Å and 1.330(3) Å, respectively. These bond parameters represent an elongation of the N–N bond and a shortening of the N–O bond compared to hyponitrite salts (Table 1). The shorter N–O bond distance is reflected by the vibrational properties of the complex, with the antisymmetric N–O stretching vibration reported at 1087 cm^−1^, which is around 50 cm^−1^ higher in energy compared to sodium *trans*‐hyponitrite. Another interesting result from the structural studies is that the Fe–O bond is approximately 0.1 Å shorter than the Fe–N bond (see Table 1), suggesting that O‐bound hyponitrite is preferred at Fe centers. EPR spectroscopy and SQUID magnetometry data revealed that [Fe_2_(NO)_4_(μ‐bdmap)]_2_(κ^4^‐N_2_O_2_) shows weak magnetic coupling between the four {Fe(NO)_2_}^9^ (*S* = 1/2) centers, likely occurring through the bridging *trans*‐hyponitrite ligand. A major limitation in using this complex to provide insights into FNOR chemistry is that the Fe centers in [Fe_2_(NO)_4_(μ‐bdmap)]_2_(κ^4^‐N_2_O_2_) are DNICs, which are electronically quite distinct compared to typical non‐heme Fe centers [1]. Overall, [Fe_2_(NO)_4_(μ‐bdmap)]_2_(κ^4^‐N_2_O_2_) represents only the second structurally characterized Fe‐hyponitrite complex reported in the literature.

#### 3.1.8 Coordination of Hyponitrite to [Fe_2_(BMPA‐PhO)_2_(OTf)_2_]

As the electronic structure of DNICs is distinct to that of the Fe centers of FNORs, it is also of interest to study and prepare non‐heme Fe‐hyponitrite complexes. Recently, we reported the third structurally characterized Fe‐hyponitrite complex. We reacted the non‐heme iron complex [Fe^II^_2_(BMPA‐PhO)_2_(OTf)_2_] (BPMA‐PhO = (2‐((bis(pyridin‐2‐ylmethyl)amino)methyl)phenolate) with sodium *trans*‐hyponitrite to obtain [{Fe^II^_2_(BMPA‐PhO)_2_}_2_(μ‐N_2_O_2_)](OTf)_2_ (Figure 2 and Scheme 4C) [78]. The structure of [{Fe^II^_2_(BMPA‐PhO)_2_}_2_(μ‐N_2_O_2_)]^2+^ was determined by single‐crystal X‐ray diffraction, revealing a *trans*‐hyponitrite ligand bound by four Fe centers (Figure 2). Interestingly, the *trans*‐hyponitrite ligand is bound in a different arrangement compared to that of the DNIC complex described above. The N–N and N–O bond lengths for the hyponitrite ligand in [{Fe^II^_2_(BMPA‐PhO)_2_}_2_(μ‐N_2_O_2_)]^2+^ are 1.257(8) Å and 1.358(6) Å, respectively (Table 1). These bond metrics are similar to those of sodium *trans*‐hyponitrite. IR spectroscopy revealed a vibrational frequency of 1070 cm^−1^ for the antisymmetric N–O stretch, which is similar to that of the DNIC complex described above. This is surprising, as the N–O bond lengths in [{Fe^II^_2_(BMPA‐PhO)_2_}_2_(μ‐N_2_O_2_)]^2+^ are similar to sodium *trans*‐hyponitrite and do not appear to be shortened, as observed for the DNIC complex. Additionally, the Fe–O bond distance is approximately 0.2 Å shorter than the Fe–N bond distance for the hyponitrite ligand (Table 1). This is similar to the DNIC complex and indicates that Fe^II^ binds stronger to oxygen in the hyponitrite ligand. As is the case for most metal‐hyponitrite complexes, the addition of acid to [{Fe^II^_2_(BMPA‐PhO)_2_}_2_(μ‐N_2_O_2_)]^2+^ leads to N_2_O release. But interestingly, the oxidation of two of the ferrous iron centers in this tetra‐iron complex also leads to the formation of N_2_O. This indicates that the Lewis acidity of the Fe centers is important for Fe‐hyponitrite stability, with a more Lewis acidic Fe center leading to the decomposition of hyponitrite. This is mechanistically relevant for FNORs, as NO reduction by the direct coupling or the semireduced mechanism generates either a diferric or mixed valent (Fe^II^/Fe^III^) hyponitrite intermediate. Here, the oxophilicity and Lewis acidity of the ferric iron then provides the driving force for N_2_O release, without the need for hyponitrite protonation.

This was further investigated by reaction of sodium *trans*‐hyponitrite with the more Lewis acidic iron center of [Fe^II^(TPA)(CH_3_CN)_2_](OTf)_2_ (TPA = tris(2‐pyridylmethyl)amine), which leads to hyponitrite cleavage to produce [Fe^II^_2_(TPA)_2_(NO)_2_](OTf)_2_ [78, 133]. How [Fe^II^(TPA)(CH_3_CN)_2_](OTf)_2_ is able to facilitate the cleavage of the N–N bond rather than N–O bond of the hyponitrite ligand remains unclear. Overall, these results provide valuable insights into the stability of hyponitrite at non‐heme Fe centers. A major limitation of this work; however, is that the Fe‐hyponitrite complex features a hyponitrite ligand bound by four Fe centers, whereas in FNORs, only two Fe centers are expected to generate the key Fe‐hyponitrite intermediate. Furthermore, the initial binding mode of the hyponitrite intermediate in FNORs is expected to be N‐bound at the Fe centers in a *cis*‐fashion, followed by rearrangement(s) of the hyponitrite binding mode [77]. Further work is necessary to truly model the hyponitrite intermediate(s) of FNORs.

### 3.2 Comparison of Fe‐Hyponitrite With Select Ni‐ and Cu‐Hyponitrite Complexes

Besides Fe, several other transition metals have been shown to form stable metal‐hyponitrite complexes, many of which were reviewed recently by Hayton and coworkers [11]. Many of these transition metals form different binding modes with the hyponitrite ligand than what has been observed for Fe‐hyponitrite systems. Although there are not yet enough transition metal‐hyponitrite complexes in the literature to fully discern whether certain binding modes are preferred for certain transition metal centers, we thought it would be useful to discuss a few different metal‐hyponitrite binding modes. Here, we discuss three transition metal‐hyponitrite complexes: [Ni(dppf)(*η* ^2^‐O_2_N_2_)] (dppf = 1,1’‐bis(diphenylphosphino)ferrocene), [KLNi_2_(N_2_O_2_)] (L = (3R)‐3,5‐bis(((4‐((2,6‐diisopropylphenyl)‐l2‐azaneyl)pentan‐2‐yl)‐l2‐azaneyl)methyl)‐1l2,2l2‐pyrazolidine), and [{Cu^II^(TPA)}_2_(μ‐N_2_O_2_)]^2+^ [124, 129, 131].

**SCHEME 5:**
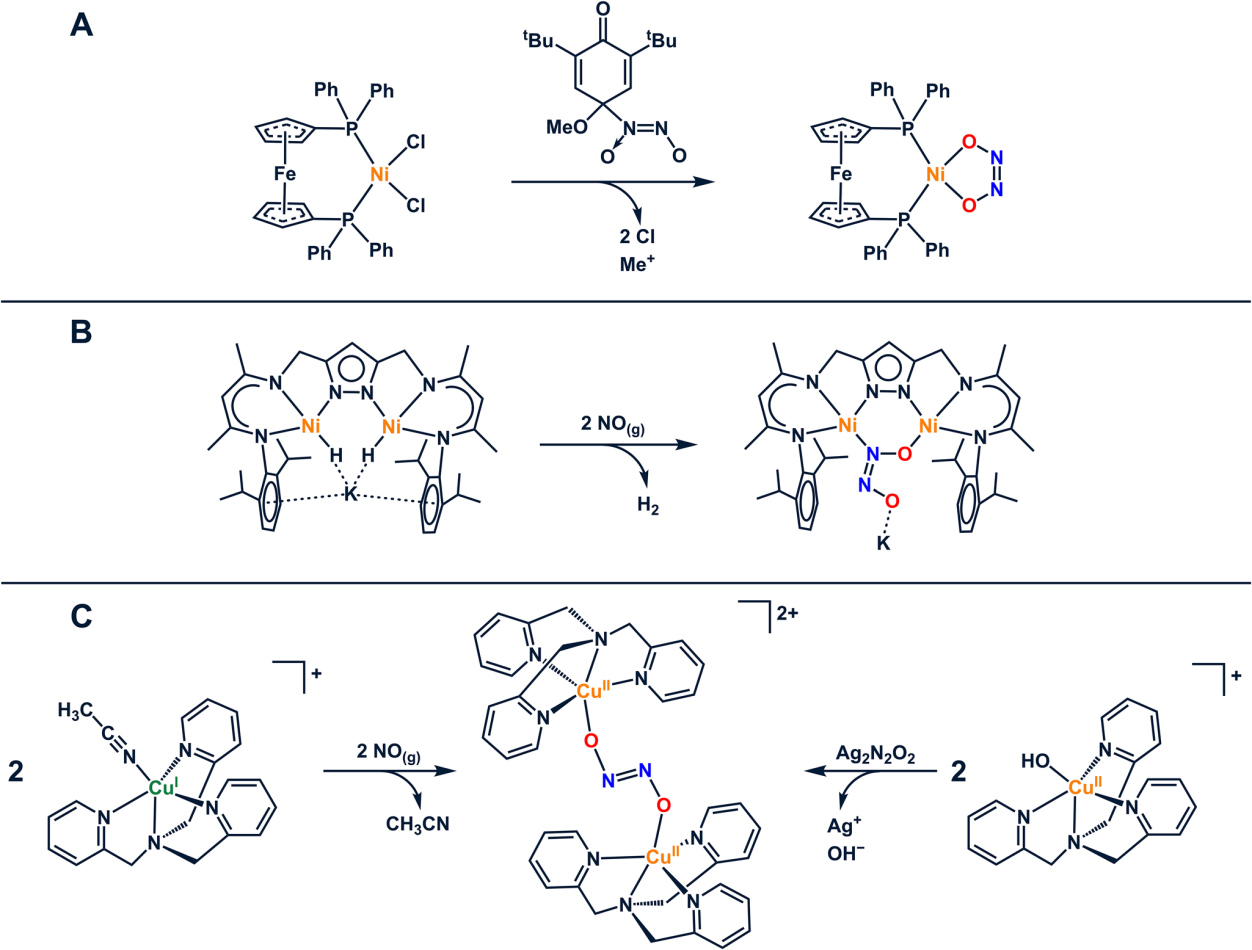
Synthetic routes used to prepare [Ni(dppf)(*η* ^2^‐O_2_N_2_)] (panel A), [KLNi_2_(N_2_O_2_)] (panel B), and [{Cu^II^(TPA)}_2_(μ‐N_2_O_2_)]^2+^ (panel C).

The preparation of [Ni(dppf)(*η* ^2^‐O_2_N_2_)] is rather unique, requiring the Umpolung reactivity of a diazeniumdiolate compound to form a *cis*‐hyponitrite complex (Scheme 5A), the structure of which was confirmed by single‐crystal X‐ray diffraction [129]. The formation of *cis*‐hyponitrite transition metal complexes is quite rare, but we note that as a d^8^ metal, nickel has a propensity to form square planar complexes, likely stabilizing the *cis*‐hyponitrite ligand. To the best of our knowledge, only d^8^ metals with a square planar geometry have been observed to form mononuclear transition metal *cis*‐hyponitrite complexes. Whether iron, a metal that often adopts octahedral ligand environments, can stabilize a *cis*‐hyponitrite ligand remains to be seen. The synthetic route for forming [Ni(dppf)(*η* ^2^‐O_2_N_2_)] using a diazeniumdiolate compound may be a good route for exploring the reactivity of *cis*‐hyponitrite at other transition metal centers such as iron.

Another example of a Ni‐hyponitrite complex was recently reported by Meyer and coworkers, prepared via a reductive coupling of NO at a dinickel complex (Scheme 5B) to form [KLNi_2_(N_2_O_2_)] [131]. Here, another *cis*‐hyponitrite nickel complex is formed as confirmed by single‐crystal X‐ray diffraction. In this case, one of the nickel centers of the dinickel complex binds one of the oxygen atoms of hyponitrite whereas the other nickel center is bound to one of the nitrogen atoms. This binding mode is quite interesting, as it resembles one of the proposed intermediate binding modes of hyponitrite in NO reduction by FNORs and model complexes [76, 77, 83]. It is possible that steric clash from the dipp (2,6‐diisopropylphenyl) arms of the ligand may play a role in favoring the binding of the hyponitrite ligand in this unusual geometry. Additionally, a potassium ion is associated with the unbound negatively charged oxygen of the hyponitrite ligand, which likely helps stabilizing this unique binding mode. Strategies for forming a hyponitrite adduct with this binding mode in diiron complexes remain underexplored.

Finally, it is interesting to discuss the formation of [{Cu^II^(TPA)}_2_(μ‐N_2_O_2_)]^2+^, recently reported by Karlin and coworkers [124]. [{Cu^II^(TPA)}_2_(μ‐N_2_O_2_)]^2+^ can either be formed from the Cu^I^ complex with NO gas or from the Cu^II^ complex with Ag_2_N_2_O_2_. Both routes lead to the formation of a *trans*‐hyponitrite ligand bound by a copper center at each of the negatively charged oxygen atoms, bridging between two Cu^II^(TPA) units (Scheme 5C). This structure was also confirmed by single‐crystal X‐ray diffraction. This binding mode resembles that of [Fe^III^(OEP)]_2_(μ‐N_2_O_2_) [101]. Interestingly, attempts to form a similar complex with [Fe^II^(TPA)(CH_3_CN)_2_](OTf)_2_ and sodium *trans*‐hyponitrite lead to hyponitrite cleavage instead, and formation of [Fe^II^_2_(TPA)_2_(NO)_2_](OTf)_2_ [78, 133]. This is a rather puzzling result. It is postulated that a more Lewis acidic metal center will lead to hyponitrite cleavage. One would expect though that with the same ligand scaffold, a Cu^II^ center would be more Lewis acid than an Fe^II^ center. Why the formation of a stable metal‐hyponitrite complex is observed for copper but not iron in this case remains unclear and further studies are needed to understand the principles governing this reactivity.

## 4 Conclusions and Future Outlooks

Hyponitrite has been proposed as the key intermediate during NO reduction, following reductive N–N bond formation between two NO molecules, in NOR enzymes. Despite extensive experimental and spectroscopic efforts; however, the key hyponitrite intermediate(s) in NOR enzymes have yet to be observed. So far, only computational work has provided insights into the highly reactive hyponitrite intermediates formed after NO coupling [30, 31, 32, 33, 54, 55, 71, 76, 77, 83]. Synthetic Fe‐based complexes appear to be the ideal target to model and study the properties of the proposed Fe‐hyponitrite intermediate(s) in NORs. Although hyponitrite has been studied for over a century, the field of iron‐hyponitrite chemistry is still in its infancy. To date, only three crystal structures of Fe‐hyponitrite complexes have been reported, as described above. These compounds have provided useful, but limited, insights into the behavior of hyponitrite at Fe centers, as they do not model the structures of the proposed hyponitrite intermediates of NORs well. Furthermore, each of the structurally characterized Fe‐hyponitrite species contains the *trans* isomer of hyponitrite. For further insights into NOR chemistry, characterization and reactivity of *cis*‐hyponitrite with Fe centers is highly desirable, as *cis*‐hyponitrite represents the more likely intermediate in NO reduction by NORs [1]. A major hinderance to studying the coordination chemistry of *cis*‐hyponitrite with metal centers is that there are no commercially available salts of this isomer. Further complicating matters is the fact that the only known synthetic routes to *cis*‐hyponitrite salts are solid‐state reactions that require specialized equipment such as a tube furnace [58] or a ball milling apparatus [121]. Commercialization of *cis*‐hyponitrite or further reaction development toward the preparation of *cis*‐hyponitrite salts would be highly beneficial for studying the biologically‐relevant coordination chemistry of hyponitrite, and understanding the reactivity and behavior of *cis*‐hyponitrite as an intermediate in NOR chemistry.

## Author Contributions

The manuscript was written through contributions of all authors, who all have given approval to the final version of the manuscript.

## Conflicts of Interest

The authors declare no conflict of interest.
